# Food Allergy Perceptions and Health-Related Quality of Life in a Racially Diverse Sample

**DOI:** 10.3390/children5060070

**Published:** 2018-06-06

**Authors:** Alicia Toeruna Widge, Elizabeth Flory, Hemant Sharma, Linda Jones Herbert

**Affiliations:** 1 National Institute of Allergy and Infectious Disease, National Institutes of Health, Bethesda, MD 20814, USA; 2 School of Medicine and Health Sciences, The George Washington University, Washington, DC 20052, USA; lflory@gwmail.gwu.edu (E.F.); HSharma@childrensnational.org (H.S.); LHerbert@childrensnational.org (L.J.H.); 3 Division of Allergy and Immunology, Children’s National Health System, Washington, DC 20010, USA

**Keywords:** food allergy, race, ethnicity, health related quality of life

## Abstract

This study examined caregiver perceptions of risk of food allergen exposure, and food allergy severity, worry, and health-related quality of life, and identified variations by race/ethnicity. Given the lack of data on racial/ethnic background in research on the psychosocial impacts of food allergy, this study meets a pressing need for research regarding food allergy-related experiences among diverse populations. This study found there were significant differences in perceived risk of allergen exposure among racial/ethnic groups with Asian Americans reporting significantly higher perceived risk of allergen exposure than Hispanic, Caucasian, and African American caregivers. There were no significant differences in food allergy severity, food allergy worry, or health-related quality of life among racial/ethnic groups; however, variability among racial/ethnic groups was apparent. Data may inform screening, counseling, and education practices for families from diverse backgrounds and aid in hypothesis generation for future research.

## 1. Introduction

Childhood food allergy prevalence is estimated at 8% in the United States and has risen at a rapid rate over the past two decades [1,2]. Food allergy has a high economic cost, including direct medical costs and indirect costs related to lost labor productivity and allergen-free foods; these costs may disproportionately affect minority populations [3]. Although clinicians are treating an increasingly diverse patient population, few studies have examined racial/ethnic disparities in food allergy-related psychosocial functioning [2,4,5]. Prior research has shown that the impact of food allergies on quality of life varies by age, gender, allergic reaction history, number of food allergies, and co-existing atopic disease; however, little is known about the impact of race/ethnicity [6,7]. Prior research on racial and ethnic disparities in food allergy has shown differences in phenotypes and health care utilization [8]. Furthermore, even when controlling for parent income and education level, African Americans with food allergies have higher rates of food insecurity and difficulty affording medications and Hispanic patients have more difficulty affording follow up care compared to Caucasians [9]. These studies indicate that minority children are at higher risk of adverse outcomes than Caucasian children, which is likely to affect their quality of life and may affect caregiver’s overall worry and perceptions of their children’s food allergy severity and risk of allergen exposure. Our aim is to explore food allergy perceptions and health-related quality of life (HRQoL) in a diverse sample with the goal of aiding in future hypothesis generation regarding the impact of race/ethnicity on psychosocial functioning.

## 2. Materials and Methods

One hundred and three English-speaking caregivers of children aged 0–18 years with at least one diagnosed food allergy, recruited from an urban, mid-Atlantic Food Allergy Clinic within an academic medical institution, completed an online survey about food allergy-related psychosocial functioning. Providers identified eligible food allergy patients at clinic appointments from March 2014 to June 2016; parents were then emailed an invitation to complete the clinical screener and a link to a REDCap (Research Electronic Data Capture) survey, which is a secure online survey and data collection tool [10]. A waiver of documentation of consent was approved for this project. Participants did not receive incentives. All study procedures were IRB-approved by the authors’ institution on 23 October 2013 (Protocol # 00004143).

For each food allergy, caregivers rated their child’s risk of exposure to the food on a 4-point Likert scale, with higher values indicating greater risk. Caregivers rated the severity of each food allergy and their worry about the food allergy on 100-point visual analog scales, with higher values indicating greater severity and worry. A mean risk of exposure score, perceived severity score, and worry score were calculated by averaging the individual food allergy exposure scores, severity scores, and worry scores for each participant. HRQoL was assessed by the Food Allergy Quality of Life—Parental Burden (FAQL-PB), a 17-item measure which assesses the impact of food allergy on caregivers’ daily lives on a 7-point Likert scale [11]. A total score is derived by averaging the items with lower scores indicating better HRQoL.

## 3. Results

**Figure 1:**
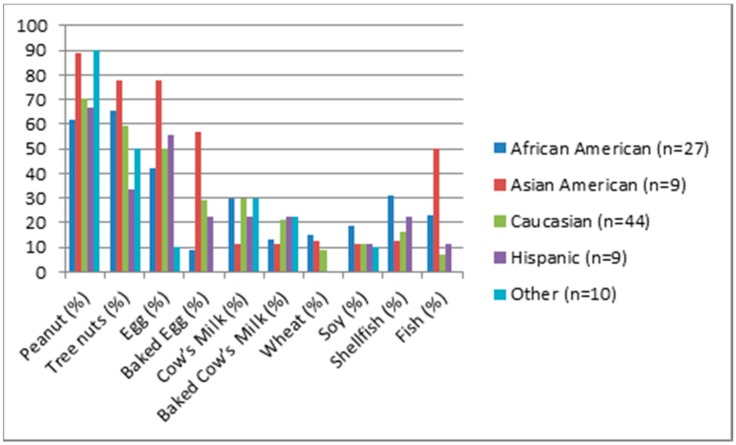
Food Allergy Frequency by Race/Ethnicity (*n* = 99). Note: Four participants declined to provide information regarding race/ethnicity. Asian Americans reported more fish ALLERGY diagnoses than other race/ethnicities *p*-value < 0.05. All other results *p* > 0.05.

Approximately 34% of all potential participants (N = 303) completed the survey. The final sample of 103 caregivers included African Americans (26%), Asian Americans (9%), Caucasians (43%), Hispanics (9%), and participants who classified themselves as an “other” race/ethnicity (10%). Four participants (4%) declined to provide information on race/ethnicity. Most caregivers self-identified as mothers (92%, *n* = 95), followed by fathers (7%, *n* = 7) and other relative (1%, *n* = 1). Mean child age was 5.28 years (*SD* = 4.35). Caregivers reported a mean of 2.85 food allergies (*SD* = 1.95). There were no significant differences in overall number of food allergies or individual food allergy diagnoses among racial/ethnic groups, *p* > 0.05, with the exception of fish allergy, which was over-represented by Asian Americans (see Figure 1).

Mean perceived allergen exposure risk was 1.38 (*SD* = 0.56), mean perceived allergen severity was 61.66 (*SD* = 23.12), mean food allergy-related worry was 65.86 (*SD* = 25.06), and mean HRQoL was 1.83 (*SD* = 1.17). Caregivers perceived varying risk of exposure, severity, and worry depending on individual allergens; however, these results were not statistically significant (See Table 1). Child age was correlated with caregivers’ overall perceived food allergy severity, *r* = 0.25, *p* < 0.05; caregivers of older children reported greater food allergy severity. Child age was not significantly related to risk of allergen exposure, food allergy-related worry, or HRQoL, *ps* > 0.05.

A univariate ANOVA controlling for age indicated that there were significant differences in perceived risk of allergen exposure among racial/ethnic groups, F(4, 93) = 3.09, *p* < 0.05. Post-hoc analyses indicated that Asian Americans reported a significantly higher overall perceived risk of allergen exposure than Hispanic, Caucasian, and African American caregivers, *ps* < 0.05 (See Table 2). After controlling for age, there were no significant differences in food allergy severity, food allergy worry, or HRQoL among racial/ethnic groups; however, variability among racial/ethnic groups was apparent. Non-Caucasian caregivers overall reported the highest food allergy severity and worry. Hispanic caregivers reported the highest perceived severity (*M* = 71.56, *SD* = 20.66), followed closely by African American (*M* = 70.34, *SD* = 20.08) and Asian American caregivers (*M* = 67.30, *SD* = 7.32). African American caregivers reported the most worry (*M* = 73.36 *SD* = 25.30), followed by Hispanic (*M* = 72.09, *SD* = 31.05) and Asian American caregivers (*M* = 71.47, *SD* = 6.43). Caucasian caregivers reported lower levels of perceived severity (*M* = 58.11 *SD* = 20.65) and worry (*M* = 63.50, *SD* = 22.11). Caregivers who classified themselves as an “other” race/ethnicity reported the lowest perceived severity (*M* = 54.91, *SD* = 34.30) and worry (*M* = 57.09, *SD* = 31.90). Hispanic caregivers reported the worst HRQoL (*M* = 2.32, *SD* = 1.06) and African American caregivers reported the best HRQoL (*M* = 1.41, *SD* = 1.05).

## 4. Discussion

Although the sample size was small, this is one of the first studies to examine food allergy perceptions and HRQoL by race/ethnicity in a diverse population. Although our findings in this study were not significant, with the exception of Asian Americans perceiving higher risk of allergen exposure, food allergy-related psychosocial experiences may vary based on sociodemographic variables, such as race/ethnicity. In this study, African American caregivers reported the lowest perceived risk of allergen exposure, but the highest worry, and Hispanic caregivers reported the highest perceived food allergy severity and lowest HRQoL. The etiology of these differences is unclear, but there are likely a constellation of genetic, environmental, dietary, and social factors that contribute to food allergy differences among these groups.

Previous research on the psychosocial impact of food allergies often does not report data on racial/ethnic background, and where it is reported, typically >85% of the population is Caucasian; therefore, this study meets a pressing need for research regarding food allergy-related experiences among diverse populations [2]. This study provides the groundwork for larger, multi-centered studies of food allergy perceptions and HRQoL among diverse populations. Based on these results, future studies may hypothesize that caregivers from racial and ethnic minorities experience higher perceived risk, severity, and worry and worse HRQoL than Caucasians. Future studies are needed to confirm this study’s findings and elucidate why experiences differ and investigate how food allergy perceptions may impact management and outcomes. Limitations of this study include lack of data on socioeconomic status, which may be a confounding factor for differences seen in race/ethnicity; however, previous research has shown that even when controlling for income and education level, differences in access to food and medical care for patients with food allergies persist [9]. Further limitations are that these results may not be fully generalizable given it was a single-site study with a small sample size. While our sample had a comparable percentage of Hispanics and Asian Americans to our clinic population, there were a higher percentage of Caucasians and a lower percentage of African Americans, which may have resulted in sampling bias. Future studies may also consider examining the association of perceived risk, severity, worry, and HRQoL with reaction history.

Food allergies can be a financial, social, and time intensive burden on families. Clinicians should be aware of the varied impact of food allergy on daily life when working with patients of diverse backgrounds, as it may influence the way they screen, counsel, and educate families about food allergy. Helping families achieve an appropriate level of concern regarding food allergy exposure risk and worry while attending to cultural differences has the potential to impact adherence to treatment, follow up, and patients’ quality of life.

## Figures and Tables

**Table 1:** Caregiver Perceptions of Risk of Food Allergen Exposure, Food Allergy Severity, Food Allergy Worry by individual allergen (*n* = 103).

	Risk of Allergen Exposure		Food Allergy Severity		Food Allergy-Related Worry	
Specific Allergen	*Mean (SD)*	*Range*	*Mean (SD)*	*Range*	*Mean (SD)*	*Range*
Overall	1.38 (0.56)	0.0–2.8	61.66 (23.12)	4–100	65.86 (25.06)	5–100
Peanut	1.45 (0.50)	0.6–2.6	64.68 (30.44)	0–100	72.69 (26.20)	0–100
Tree nuts	1.48 (0.53)	0.0–2.4	67.26 (29.13)	0–100	73.90 (24.84)	0–100
Direct Egg	1.21 (0.73)	0.0–2.8	49.07 (25.14)	11–100	44.80 (29.39)	8–100
Baked Egg	1.59 (0.55)	0.8–2.8	67.70 (24.35)	8–100	70.21 (27.95)	6–100
Direct Milk	1.75 (0.87)	1.0–3.0	47.00 (10.34)	35–62	45.40 (20.85)	10–65
Baked Milk	1.74 (0.62)	0.6–2.8	79.78 (21.52)	26–100	76.65 (27.45)	8–100
Wheat	1.76 (0.65)	0.0–2.8	71.75 (29.32)	30–100	62.88 (41.18)	0–100
Soy	1.37 (0.73)	0.0–2.4	52.89 (25.69)	8–77	49.91 (29.45)	0–81
Shellfish	1.19 (0.78)	0.0–2.4	73.31 (25.66)	26–100	64.47 (32.83)	6–100
Fish	1.63 (0.68)	0.0–2.6	81.25 (16.43)	55–100	70.85 (27.01)	5–100

*p*-value is >0.05 for all results in Table 1.

**Table 2:** Caregiver Perceptions of Risk of Food Allergen Exposure, Food Allergy Severity, Food Allergy Worry, and Health-Related Quality of Life (HRQoL) by Race/Ethnicity (N = 99).

	Risk of Allergen Exposure		Food Allergy Severity		Food Allergy-Related Worry		Health-Related Quality of Life	
Race/Ethnicity	*Mean (SD)*	*Range*	*Mean (SD)*	*Range*	*Mean (SD)*	*Range*	*Mean (SD)*	*Range*
African American	1.15 (0.62)	0.0–2.8	70.34 (20.08)	22–100	73.36 (25.30)	5–100	1.41 (1.05)	0.0–4.1
Asian American	1.81 * (0.35)	1.2–2.2	67.30 (7.32)	60–79	71.47 (6.43)	62–78	2.07 (1.09)	0.9–3.7
Caucasian	1.48 (0.51)	0.5–2.3	58.11 (20.65)	10–100	63.50 (22.11)	15–100	2.00 (1.21)	0.1–5.5
Hispanic	1.38 (0.69)	0.3–2.4	71.56 (20.66)	38–97	72.09 (31.05)	7–100	2.32 (1.06)	1.0–3.9
Other	1.25 (0.46)	0.6–1.8	54.91 (34.30)	7–100	57.09 (31.90)	10–100	1.60 (0.80)	0.1–2.7

* *p*-value < 0.05.
